# Exploring the Relationship between Walking and Emotional Health in China

**DOI:** 10.3390/ijerph17238804

**Published:** 2020-11-27

**Authors:** Zhenjun Zhu, Hongsheng Chen, Jianxiao Ma, Yudong He, Junlan Chen, Jingrui Sun

**Affiliations:** 1 College of Automobile and Traffic Engineering, Nanjing Forestry University, Nanjing 210037, China; zhuzhenjun@njfu.edu.cn (Z.Z.); majx@njfu.edu.cn (J.M.); heyudong@njfu.edu.cn (Y.H.); sunjingrui@njfu.edu.cn (J.S.); 2 School of Architecture, Southeast University, Nanjing 210096, China; 3 School of Transportation, Southeast University, Nanjing 211189, China; 220183011@seu.edu.cn

**Keywords:** walking, emotional health, physical exercises, air pollution, health effect

## Abstract

Walking has a positive impact on people’s emotional health. However, in the case of serious air pollution, it is controversial whether walking exercise can still improve individuals’ emotional health. Using data from the 2014 wave of the China Labor-Force Dynamics Survey, this study explored the relationship between walking and emotional health with different levels of environmental pollution. The results indicated that respondents who took regular walks had better emotional health than those who did not walk regularly. For those whose main mode of physical exercise was walking, the average number of walks per week was significantly and positively correlated with their emotional health; however, the average duration of the walk had no significant impact on their emotional health. Moreover, for those whose main mode of physical exercise was walking and who lived in neighborhoods with a polluted environment, regular walking still had a positive impact on their emotional health. This suggests that even if environmental pollution is serious, walking still plays an important role in regulating individuals’ mental health. We propose that in order to promote the emotional health of residents, it is necessary to create more public spaces for outdoor activities and simultaneously increase efforts to control environmental pollution.

## 1. Introduction

Economic development has not only improved individuals’ living standards but has also created some environmental problems. For example, the issue of air pollution is becoming increasingly serious in many developing countries. The frequent occurrence of haze and other phenomena poses problems to individuals’ daily life and health [1]. Inadequate environments are likely to reduce individuals’ outdoor activities [2]. In modern societies, individuals tend to work long hours, and the work intensity is high; moreover, sedentary lifestyles are very common, with increasingly more individuals showing a lack of physical activity. All these factors affect individuals’ physical and mental health. It has been shown that physical inactivity may play an important mediating role in some physical and psychological disorders [3,4,5,6].

Many studies have demonstrated the positive effects of physical exercise on individuals’ health [7,8]. However, the health effects of physical activity have been found to slightly differ between developed and developing countries. Developed countries have a higher economic level, and more individuals have fitness and exercise habits [9]. Additionally, exercise shows more positive effects in developed countries. Conversely, developing countries are in a stage of rapid economic growth, and the consequent environmental pollution and rapid urbanization have led to reduced physical activity levels among the population [10]. During this transition from a traditional society to a modern one, many individuals have not yet developed new healthy lifestyles (e.g., daily or weekly physical exercise). This has led to differences between countries in the impact of exercise on health. China is in an era of rapid economic expansion, and the improvement of living standards has also promoted the pursuit of healthier habits.

Increasingly larger studies have explored the relationship between physical exercise and individuals’ mental health, and some have found that physical exercise does improve mental health [11,12,13,14,15,16,17,18,19,20]. For example, Netz et al. [17] determined that physical exercise has important beneficial effects on individuals’ physical and mental health. Li et al. [18] found that individuals who regularly engaged in physical activity had better mental health and better levels of personal work performance than those who were sedentary or inactive. Another study showed that physical exercise affects the processing of negative emotional information and promotes individuals’ mental health [19]. Puett et al. [20] examined questionnaire data and clinical evaluations of men and women over the age of 20 and found that those who did not exercise had a significantly higher risk of poor mood and stress than those who engaged in regular physical exercise.

With the development of living standards, more research has started to focus on the impact of walking on individuals’ mental health. In past studies, there is abundant evidence that walking promotes recovery from mental health disorders [21,22] and is an important measure to improve mental health [23,24]. Kekäläinen et al. [25] demonstrated that walking was positively associated with participants’ mental, social, and subjective health. Moreover, research found that walking-based exercise training alleviated anxiety and depression in adults, and moderate and intense walking exercise promoted lower blood pressure and improved mental health by reducing negative emotions [26,27]. In addition, in individuals who generally prefer more casual forms of exercise, such as walking, increasing walking during leisure time can reduce the generation of negative emotions [28]. In a study by Wensley and Slade [29], walking increased well-being by making participants feel more relaxed. Lin et al. [30] also found that walking was beneficial in reducing stress, suggesting that cities should provide more public spaces conducive to walking exercise.

Generally, most previous studies focused on the impact of walking on mental health, while research data on the impact factors of walking time and environment are not comprehensive. For instance, Ekkekkakis et al. [31] identified a positive association between the duration of physical activity and mental health, but did not specifically examine walking as a form exercise. Yang et al. [27] also pointed out the scarcity of data on the impact of walking time. Additionally, data on the effects of walking on mental health under different environmental conditions are not clear. Under the influence of serious air pollution and poor comfort of external environments, research on the relationship between walking and mental health has been scarce and data are not comprehensive [32]. Marselle et al. [33] determined that walking in nature had a positive effect on mental health, but did not distinguish different environmental factors. Therefore, the relationship between walking and mental health with different levels of environmental quality deserves a more in-depth analysis [24].

Thus, there are still some research gaps concerning the relationship between walking and emotional health. First, most available research results are concentrated in developed countries, while research in developing countries has been scarce. Especially in a country like China that is undergoing rapid urbanization, individuals’ living environment, physical exercise style, and mental health are changing. Second, previous studies have shown the link between walking and mental health, however, it is not clear how walking in environments with different qualities will affect emotional health. To address these knowledge gaps, this study specifically explored the link between walking and emotional health in China, and investigated whether walking frequency and duration were important for emotional health. Specifically, this study examined the effect of walking on emotional health in an environment with poor air quality. This study will contribute to a better understanding of the relationship between walking and emotional health under different environmental conditions in China. Furthermore, this case study of China will also help better understand the increasing “lifestyle–environmental health” issues in other developing countries.

## 2. Materials and Methods

### 2.1. Data

We used data from the 2014 wave of the China Labor-Force Dynamics Survey (CLDS 2014), conducted by the Centre for Social Science Survey of the Sun Yat-sen University. In this survey, respondents were selected using probability proportional to size sampling. In the present study, we used the method of continuously narrowing the sample to analyze the relationship between walking and emotional health with different levels of air pollution. First, based on all respondents, we analyzed the impact of physical exercise on emotional health. For this, after eliminating those with missing data, a total of 23,327 respondents were included in the regression analysis. In this part of the analysis, valid samples accounted for 98.87% of the total sample.

Next, to analyze the relationship between walking and emotional health, we identified the group for whom walking was the main physical exercise. In the CLDS 2014, there were 4876 respondents who regularly engaged in physical exercise, accounting for 20.67% of the total sample. Among them, 2492 reported walking as their most practiced physical exercise, accounting for 51.10% of those who exercised regularly. After eliminating those with missing information, a total of 2384 valid samples were included in the regression analysis.

Finally, we focused on the group for whom walking was the main physical exercise and who lived in neighborhoods with serious air pollution. In the CLDS 2014, there were 5694 respondents living in communities with environmental pollution, accounting for 24.52% of the total sample. Environmental pollution mainly included air, soil, water, and noise pollution. Because air pollution has the most direct impact on “walking-health”, this study further selected residents living in communities with severe air pollution. A total of 1867 individuals lived in communities with severe air pollution (very serious and relatively serious), accounting for 32.79% of the total sample. Thus, after eliminating those with missing information, 249 valid samples were included in the regression analysis.

### 2.2. Dependent Variable

The dependent variable was respondents’ emotional health. Respondents were asked the following four questions about their emotional health: (1) Have you often felt depressed in the last 4 weeks? (2) Have you often felt discouraged in the last 4 weeks? (3) Have you often felt unable to overcome difficulties in the last 4 weeks? (4) Have you often felt sad in the last 4 weeks? Respondents answered each question using a 5-point Likert-type scale ranging from 1 to 5 (1 = always, 2 = often, 3 = occasionally, 4 = seldom, 5 = no). Cronbach’s alpha for the four items was 0.89. We added up the scores of the four items to obtain the emotional health score for each respondent.

### 2.3. Independent Variables and Covariates

The independent variables comprised physical exercise types, average number of walks per week, and average walk duration. Respondents’ physical exercise types were divided into three groups: no regular physical exercise, walking, and other physical exercises (e.g., swimming, Tai Chi, mountaineering). The average number of walks per week ranged between 1 and 7, and the mean number was 5.33 (SD = 1.97). The average walk duration was between 1 and 360 min, and the mean duration was 48.56 min (SD = 33.30 min). In this study, we used individual demographic factors as covariates, which included age, self-rated physical health, marital status, and neighborhood types. Table 1 shows the summary statistics of the variables used in the regression models.

### 2.4. Statistical Analysis

Since respondents’ emotional health was a continuous variable, we adopted the linear regression method for analysis. We conducted the following three regression models, in order. Model 1 included all respondents and mainly analyzed the impact of the type of physical exercise on emotional health. Model 2 included respondents for whom walking was their primary type of physical exercise and mainly analyzed the impact of walking frequency and duration on emotional health. Model 3 included respondents for whom walking was their primary type of physical exercise and who lived in neighborhoods with serious air pollution. This last model analyzed whether walking had a significant impact on emotional health when the respondent considered the air was highly polluted. Since individuals’ emotional health is affected by many factors, a single factor or a certain type of factor will have a relatively small impact on it; thus, in this study, R^2^ and adjusted R^2^ were relatively small. However, from Model 1 to Model 3, the values of R^2^ and adjusted R^2^ gradually increased, indicating that as the sample size decreased, the independent variables explained the dependent variables to an increasingly larger extent.

## 3. Results

Table 2 shows the regression analyses results for the relationship between emotional health and walking. In Model 1, including the whole sample, the respondents who took regular walks (B = 0.362, *p* < 0.01) or engaged in other forms of physical exercise (B = 0.659, *p* < 0.01) had better emotional health than those who did not exercise regularly. In terms of individual demographic factors, age (B = 0.026, *p* < 0.01) and self-rated physical health (B = 0.684, *p* < 0.01) were significantly and positively and associated with emotional health. Compared with single respondents, married respondents reported better emotional health (B = 0.159, *p* < 0.05), and divorced/widowed respondents (B = −0.537, *p* < 0.01) reported worse emotional health. Respondents who lived in urban neighborhoods reported poorer emotional health than those who lived in rural neighborhoods (B = −0.117, *p* < 0.01).

In Model 2, including those for whom walking was the main physical exercise, the average number of walks per week was significantly and positively correlated with emotional health (B = 0.143, *p* < 0.01), but the average walk duration had no significant impact on emotional health. In terms of individual demographic factors, age (B = 0.033, *p* < 0.01) and self-rated physical health (B = 0.740, *p* < 0.01) were significantly and positively associated with emotional health, and married respondents reported better emotional health than single respondents (B = 0.438, *p* < 0.10).

In Model 3, including those for whom walking was the main physical exercise and who lived in neighborhoods with serious air pollution, the average number of walks per week was significantly associated with emotional health (B = 0.207, *p* < 0.05). That is, for respondents from neighborhoods with serious air pollution, regular walking still had a positive impact on emotional health. In terms of individual demographic factors, self-rated physical health (B = 0.955, *p* < 0.01) was significantly and positively associated with emotional health, and married respondents reported better emotional health than single respondents (B = 2.122, *p* < 0.01).

## 4. Discussion

Emotional health reflects a person’s psychological state and is not only affected by the internal psychological cognitive structure [34] (e.g., long-term outlook on life, values, and attitudes towards life [35,36]) but also by the external environment (e.g., relaxing natural environment [37,38]). Further, individuals can adjust their mental state by choosing different exercise methods and release stress through exercise [39]. As a slow-paced exercise mode, walking has an obvious regulating effect on emotional health. Individuals can organize their thoughts through easy walking activities, which may also help them find solutions to their difficulties.

In this study, we found that walking had a positive effect on emotional health, which was consistent with previous research [24,25,26,27,28,29]. A potential explanation for this could be that when walking, individuals may come in contact with nature, which soothes the body and mind. Relevant studies have pointed out that after outdoor group walks, the perception of restorativeness and naturalness influence each other, thus enhancing the positive emotional experience [24]. In addition, walking is an enjoyable form of exercise that can alleviate individuals’ anxiety [40,41,42] and depression [8,43], and enhance their mental well-being [44,45,46]. Wensley et al. [29] described walking as a kind of joy and considered it a source of positive emotions, and Kekäläinen et al. [25] found that walking was associated with better psychological well-being.

In addition, we found that the length of walking time had no significant effect on emotional health. Namely, regardless of the walk duration, walking improved emotional health consistently. On the one hand, we think this may be related to the surrounding environment. For example, when one first enters a park, one will feel the fresh air and experience physical and mental comfort; however, after a period of time, the experience might become normal or ordinary. Similar theories can be found in previous research [47]. Different lengths of time spent in an environment had almost the same effect on improving self-esteem, and the experience of long- and short-term exposure was almost the same [48]. On the other hand, it may be that individuals’ perception of the impact of walking time was not obvious and was easily interfered with by other factors. Marselle et al. [24] mentioned that the perception of walking intensity precedes that of walking duration.

Moreover, we found that even for residents of neighborhoods with poor air quality, the positive impact of walking on emotional health was maintained. This is somewhat different from the findings of previous studies. For example, Gu et al. [49] found that air pollution had a negative impact on mental health. Moreover, Yu et al. [50] reported that air pollution seriously hindered individuals’ daily physical exercise (e.g., walking, biking). Even in our opinion, walking in an environment with poor air quality is uncomfortable. A reason why previous research conclusions and common sense are different from our finding could be that past research did not consider the impact of walking time on emotional health under conditions of poor air quality. In the same length of time, the positive impact of short distance walking would be far greater than the negative impact of poor air quality. Thus, air quality is only one influencing factor in the walking environment, and this single factor alone is not enough to affect the positive impact of walking on emotional health. This explanation is consistent with the results of Han et al. [32].

Based on our results, in order to improve the emotional health of Chinese individuals, we make the following suggestions. First, individuals should plan their time and do special walking exercises and take short commutes by foot. Second, the community needs to promote individuals’ passion for walking, for example, by setting up walking groups, setting up WeChat step count competitions, integrating community trails, adding green plants, and improving the community walking environment. Third, the government should issue policies (e.g., adding more walking roads in urban planning) and implement campaigns to promote walking (e.g., publicizing the effects of walking on health in the form of lectures to enhance awareness). Fourth, increasing urban green space is an important measure to promote residents’ engagement in outdoor activities. Judging from the experience of developed countries such as the Netherlands [51,52], green urban suburbs and community parks have a direct impact on increasing residents’ outdoor activities. Due to the dense urban residential area layout in China, green spaces inside the cities have often not been protected or reserved, which limits the possibilities for residents to perform outdoor activities nearby. Controlling the urban growth boundary and creating green spaces inside the city and in the community is of great significance to promoting the physical and mental health of residents.

The relationship of physical exercise, mental health, and the natural environment is very complex and deserves more in-depth research. The impact of walking on emotional health is related not only to the amount of time spent walking but also to the intensity of the walking exercise. However, since the data used in this study did not contain information on exercise intensity, research on the impact of exercise intensity on the emotional health of walkers needs to be conducted in the future. Another limitation of this study was the lack of accurate measurement of air quality. In the future, more accurate and higher-quality measurements of natural environmental factors are required to study the mental health effects of physical exercise for different types of individuals under different natural environmental conditions. In addition, there is a difference between the short- and long-term impact of physical exercise on mental health [53]. In the future, tracking survey data may thus be used to analyze the impact of exercise on individuals’ mental health.

## 5. Conclusions

Walking has an important impact on the emotional health of individuals. This study found that respondents who engaged in regular walks or other forms of physical exercise had better emotional health than those who did not exercise regularly. The average number of walks per week was significantly and positively associated with emotional health, however, the average walk duration had no significant impact on the emotional health of respondents. This study further revealed that for respondents living in neighborhoods with serious air pollution, regular walking still had a positive impact on their emotional health. Thus, encouraging residents to take regular walks is of great significance for regulating their mental health.

## Figures and Tables

**Table 1:** Summary statistics of study variables.

Variables	Mean/%	SD
Emotional health (4–20)	16.26	3.26
Physical exercise types (%)		
No physical exercise regularly	79.38	
Walking	10.55	
Other physical exercises	10.07	
Age (years old)	42.91	14.51
Self-rated physical health (1–5)	3.69	0.99
Marital status (%)		
Single	14.29	
Married	81.04	
Widowed or divorced	4.67	
Neighborhood types (%)		
Rural neighborhood	61.35	
Urban neighborhood	38.65	

**Table 2:** Regression analysis results for the relationship between emotional health and walking.

Independent Variables	Model 1		Model 2		Model 3	
	Coefficient	SE	Coefficient	SE	Coefficient	SE
Physical exercise types (ref: No physical exercise regularly)						
Walking	0.362 ***	(0.070)				
Other physical exercises	0.659 ***	(0.072)				
Average number of walks per week			0.143 ***	(0.033)	0.207 **	(0.100)
Average duration per walk			0.001	(0.002)	0.005	(0.005)
Age	0.026 ***	(0.002)	0.033 ***	(0.006)	0.007	(0.019)
Self-rated physical health	0.684 ***	(0.022)	0.740 ***	(0.070)	0.955 ***	(0.215)
Marital status (ref: Single)						
Married	0.159 **	(0.075)	0.438 *	(0.254)	2.122 ***	(0.671)
Widowed or divorced	−0.537 ***	(0.124)	−0.005	(0.366)	1.447	(1.230)
Neighborhood type (ref: Rural neighborhood)						
Urban neighborhood	−0.117 ***	(0.045)	0.036	(0.137)	−0.411	(0.413)
Constants	12.457 ***	(0.126)	11.095 ***	(0.432)	10.079 ***	(1.308)
Number of samples	23,327		2384		249	
R^2^	0.047		0.067		0.152	
adj. R^2^	0.047		0.064		0.128	
Log likelihood	−60,067.978		−6045.536		−616.920	

SE: standard error (in parentheses). * *p* < 0.10, ** *p* < 0.05, *** *p* < 0.01.

## References

[1] Dhutia H., Sharma S. (2015). Playing it safe: Exercise and cardiovascular health. Practitioner.

[2] Atkinson K., Lowe S., Moore S. (2016). Human development, occupational structure and physical inactivity among 47 low and middle income countries. Prev. Med. Rep..

[3] Malinauskiene V., Malinauskas R., Malinauskas M. (2019). Leisure-time physical inactivity and psychological distress in female-dominated occupations in lithuania. Women Health.

[4] Cannioto R., Etter J.L., LaMonte M.J., Ray A.D., Joseph J.M., Al Qassim E., Eng K.H., Moysich K.B. (2018). Lifetime physical inactivity is associated with lung cancer risk and mortality. Cancer Treat. Res. Commun..

[5] Chien L.-C., Li X., Staudt A. (2017). Physical inactivity displays a mediator role in the association of diabetes and poverty: A spatiotemporal analysis. Geospat. Health.

[6] Xu G., Sui X., Liu S., Liu J., Liu J., Li Y., Huang S., Wang Z., Blair S.N. (2014). Effects of insufficient physical activity on mortality and life expectancy in jiangxi province of china, 2007–2010. PLoS ONE.

[7] He N., Ye H. (2020). Exercise and hyperlipidemia. Adv. Exp. Med. Biol..

[8] Park S.D., Yu S.H. (2015). The effects of nordic and general walking on depression disorder patients’ depression, sleep, and body composition. J. Phys. Sci..

[9] Yi J., Horton P. (2015). The integration of three types of lands: A new approach to the provision of public sport and recreation areas in china. Int. J. Hist. Sport.

[10] Day K. (2018). Physical environment correlates of physical activity in developing countries: A review. J. Phys. Act. Health.

[11] Ten Have M., de Graaf R., Monshouwer K. (2011). Physical exercise in adults and mental health status findings from the netherlands mental health survey and incidence study (nemesis). J. Psychosom. Res..

[12] Noh E., Kim J., Kim M., Yi E. (2020). Effectiveness of sabang-dolgi walking exercise program on physical and mental health of menopausal women. Int. J. Environ. Res. Public Health.

[13] Jun K.M. (2017). The relationships between physical activity participation, exercise identity, and mental health among university students. Korean J. Sport Stud..

[14] Pascoe M., Bailey A.P., Craike M., Carter T., Patten R., Stepto N., Parker A. (2020). Physical activity and exercise in youth mental health promotion: A scoping review. BMJ Open Sport Exerc. Med..

[15] Amatriain-Fernandez S., Murillo-Rodriguez E.S., Gronwald T., Machado S., Budde H. (2020). Benefits of physical activity and physical exercise in the time of pandemic. Psychol. Trauma.

[16] Cherubal A.G., Suhavana B., Padmavati R., Raghavan V. (2019). Physical activity and mental health in india: A narrative review. Int. J. Soc. Psychiatr..

[17] Netz Y., Wu M.-J., Becker B.J., Tenenbaum G. (2005). Physical activity and psychological well-being in advanced age: A meta-analysis of intervention studies. Psychol. Aging.

[18] Li G.S.-F., Lu F.J.H., Wang A.H.-H. (2009). Exploring the relationships of physical activity, emotional intelligence and health in taiwan college students. J. Exerc. Sci. Fit..

[19] Qiu F.H., Peng W.W., Li M.M., Zhang L.L., Zhu H., Tan X.Y., Li H., Zhang J. (2019). Effects of physical exercise on negative emotional susceptibility in young adult females: An event-related potential study. Brain Res..

[20] Puett R., Teas J., Espana-Romero V., Artero E.G., Lee D.C., Baruth M., Sui X., Montresor-Lopez J., Blair S.N. (2014). Physical activity: Does environment make a difference for tension, stress, emotional outlook, and perceptions of health status?. J. Phys. Act. Health.

[21] Kim Y., Lee Y.-M., Cho M., Lee H. (2019). Effect of a pedometer-based, 24-week walking intervention on depression and acculturative stress among migrant women workers. Int. J. Environ. Res. Public Health.

[22] Browne J., Penn D.L., Battaglini C.L., Ludwig K. (2016). Work out by walking: A pilot exercise program for individuals with schizophrenia spectrum disorders. J. Nerv. Ment. Dis..

[23] Kelly P., Williamson C., Niven A.G., Hunter R., Mutrie N., Richards J. (2018). Walking on sunshine: Scoping review of the evidence for walking and mental health. Br. J. Sports Med..

[24] Marselle M.R., Irvine K.N., Lorenzo-Arribas A., Warber S.L. (2014). Moving beyond green: Exploring the relationship of environment type and indicators of perceived environmental quality on emotional well-being following group walks. Int. J. Environ. Res. Public Health.

[25] Kekäläinen T., Freund A.M., Sipilä S., Kokko K. (2019). Cross-sectional and longitudinal associations between leisure time physical activity, mental well-being and subjective health in middle adulthood. Appl. Res. Qual. Life.

[26] Shin Y. (1999). The effects of a walking exercise program on physical function and emotional state of elderly korean women. Public Health Nurs..

[27] Yang C.H., Conroy D.E. (2018). Feasibility of an outdoor mindful walking program for reducing negative affect in older adults. J. Aging Phys. Act.

[28] Mondal A., Bhat C.R., Costey M.C., Bhat A.C., Webb T., Magassy T.B., Pendyala R.M., Lam W.H.K. (2020). How do people feel while walking? A multivariate analysis of emotional well-being for utilitarian and recreational walking episodes. Int. J. Sustain. Transp..

[29] Wensley R., Slade A. (2012). Walking as a meaningful leisure occupation: The implications for occupational therapy. Br. J. Occup. Ther..

[30] Lin W., Chen Q., Jiang M., Tao J., Liu Z., Zhang X., Wu L., Xu S., Kang Y., Zeng Q. (2020). Sitting or walking? Analyzing the neural emotional indicators of urban green space behavior with mobile eeg. J. Urban Health.

[31] Ekkekakis P. (2003). Pleasure and displeasure from the body: Perspectives from exercise. Cogn. Emot..

[32] Han K.-T. (2020). The effect of environmental factors and physical activity on emotions and attention while walking and jogging. J. Leis. Res..

[33] Marselle M.R., Warber S.L., Irvine K.N. (2019). Growing resilience through interaction with nature: Can group walks in nature buffer the effects of stressful life events on mental health?. Int. J. Environ. Res. Public Health.

[34] Bunce D., Tzur M., Ramchurn A., Gain F., Bond F.W. (2008). Mental health and cognitive function in adults aged 18 to 92 years. J. Gerontol..

[35] Hagerty B.M., Williams R.A., Coyne J.C., Early M.R. (1996). Sense of belonging and indicators of social and psychological functioning. Arch. Psychiatr. Nurs..

[36] Rissanen T., Viinamki H., Honkalampi K., Lehto S.M., Koivumaa-Honkanen H. (2011). Long term life dissatisfaction and subsequent major depressive disorder and poor mental health. BMC Psychiatry.

[37] James P., Banay R.F., Hart J.E., Laden F. (2015). A review of the health benefits of greenness. Curr. Epidemiol. Rep..

[38] Chen H., Liu Y., Zhu Z., Li Z. (2017). Does where you live matter to your health? Investigating factors that influence the self-rated health of urban and rural chinese residents: Evidence drawn from chinese general social survey data. Health Qual. Life Outcomes.

[39] Chekroud S.R., Gueorguieva R., Zheutlin A.B., Paulus M., Krumholz H.M., Krystal J.H., Chekroud A.M. (2018). Association between physical exercise and mental health in 1·2 million individuals in the USA between 2011 and 2015: A cross-sectional study. Lancet Psychiatry.

[40] Lee S.C. (2016). The effects of a regular walking program on body composition, functional fitness, and anxiety and depression in elderly women. J. Korean Soc. Integr. Med..

[41] Alparslan G.B., Akdemir N. (2010). Effects of walking and relaxation exercises on controlling hypertension. J. Aust. Tradit. Med. Soc..

[42] Edwards M.K., Rosenbaum S., Loprinzi P.D. (2018). Differential experimental effects of a short bout of walking, meditation, or combination of walking and meditation on state anxiety among young adults. Am. J. Health Promot..

[43] Song H.S. (2016). A study on the relationship among depression, walking and quality of life for the elderly—Focusing on the moderation effects of walking. J. Digit. Converg..

[44] Vert C., Gascon M., Ranzani O., Marquez S., Triguero-Mas M., Carrasco-Turigas G., Arjona L., Koch S., Llopis M., Donaire-Gonzalez D. (2020). Physical and mental health effects of repeated short walks in a blue space environment: A randomised crossover study. Environ. Res..

[45] Zurawik M.A. (2020). Socio-environmental influences on nordic walking participation and their implications for well-being. J. Outdoor Recreat. Tour. Res. Plan. Manag..

[46] Thogersen-Ntoumani C., Loughren E.A., Taylor I.M., Duda J.L., Fox K.R. (2014). A step in the right direction? Change in mental well-being and self-reported work performance among physically inactive university employees during a walking intervention. Ment. Health Phys. Act..

[47] Mackay G.J., Neill J.T. (2010). The effect of “green exercise” on state anxiety and the role of exercise duration, intensity, and greenness: A quasi-experimental study. Psychol. Sport Exerc..

[48] Barton J., Hine R., Pretty J. (2009). The health benefits of walking in greenspaces of high natural and heritage value. J. Integr. Environ. Sci..

[49] Gu H., Yan W., Elahi E., Cao Y. (2020). Air pollution risks human mental health: An implication of two-stages least squares estimation of interaction effects. Environ. Sci. Pollut. Res..

[50] Yu H., An R., Andrade F. (2017). Ambient fine particulate matter air pollution and physical activity: A longitudinal study of university retirees in beijing, china. Am. J. Health Behav..

[51] Maas J., Verheij R.A., Groenewegen P.P., De Vries S., Spreeuwenberg P. (2006). Green space, urbanity, and health: How strong is the relation?. J. Epidemiol. Community Health.

[52] Wang Y., de Groot R., Bakker F., Wörtche H., Leemans R. (2017). Thermal comfort in urban green spaces: A survey on a dutch university campus. Int. J. Biometeorol..

[53] Reiner M., Niermann C., Jekauc D., Woll A. (2013). Long-term health benefits of physical activity—A systematic review of longitudinal studies. BMC Public Health.

